# Endobronchial foreign body removal using fiberoptic bronchoscope together with gastroscope biopsy forceps: A case report

**DOI:** 10.1097/MD.0000000000017424

**Published:** 2019-10-04

**Authors:** Guangshan Hao, Lina Tang, Zhengang Ji, Jianxin Zhu, Lusu Yao

**Affiliations:** a Department of Neurosurgery; b Department of Neurological Intensive Care Unit, Liaocheng People's Hospital, Liaocheng, China.

**Keywords:** fiberoptic bronchoscope, foreign body aspiration, gastroscope biopsy forceps

## Abstract

**Rationale:**

There are many difficult cases in the clinic because of the diversity of foreign bodies. The removal of a syringe cap is not so easy because there is always no hole at the closed end.

**Patient concerns:**

A 54-year-old man suddenly developed dyspnea during his treatment in the hospital.

**Diagnoses:**

Foreign body in the left main bronchus.

**Interventions:**

The foreign body was removed using fiberoptic bronchoscope together with gastroscope biopsy forceps.

**Outcomes:**

A repeat CT showed well inflation of left lung.

**Lessons:**

The combined use of gastroscope biopsy forceps in trachea is more conducive to remove a foreign body similar to a syringe cap.

## 1 Introduction

Foreign body aspiration (FBA) is a common disease in the Respiratory Department and Intensive Care Unit, which can result in long-term respiratory symptoms or even be devastating. Despite the progress we have made in bronchoscopy technology, there are still many difficult cases in the clinic because of the diversity of foreign body (FB). Sometimes a thoracotomy is required to remove the FB for some patients causing serious complications.^[1,2]^ Taking different measures according to different cases may make the removal of FBs much easier and safer. This case report is the first to demonstrate that the combined application of bronchoscopy and gastroscopic biopsy forceps can remove some FBs which are difficult to be retrieved by using bronchoscopy alone.

## 2 Case presentation

**Figure 1:**
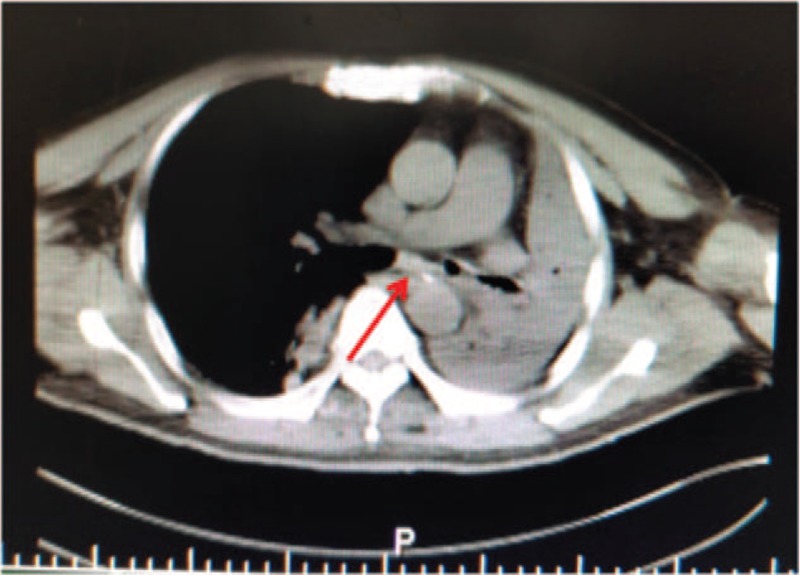
CT showed pulmonary atelectasis of the left lung and the possible of a FB in the left main bronchus.

**Figure 2:**
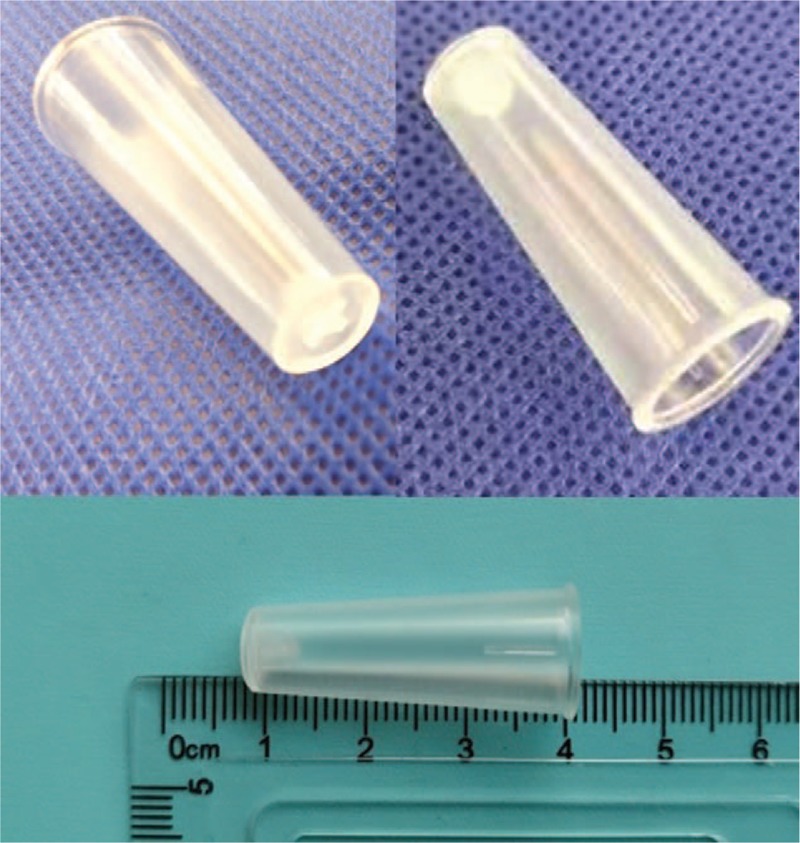
A 60 mL-syringe cap.

A 54-year-old man hospitalized because of unconsciousness after cerebral trauma suddenly developed tachypnea and cyanosis when he was undergoing oral examination employing a syringe cap. After emergency trachea intubation, he was transferred into the NICU. Physical examination was notable for the three-concave sign and the disappearance of left lung breath sounds. Computed tomography (CT) examination, done with the aid of a transport ventilator, revealed left lung atelectasis and suggested the possibility of a FB in the left main bronchus about 3 cm away from carina (Fig. 1). The FB, a hollow plastic hard object with the “+” logo at the bottom, was visualized under fiberoptic bronchoscope (Olympus LF-TP; Olympus Co., Tokyo, Japan, the external diameter 4.0 mm). It was stuck in the left main bronchus with its sidewall closely attached to the bronchus. We could only carefully clamp the small edge of the “+” mark and pulled it into the space above the tracheal carina but it could not be grasped firmly. In addition, the FB was too large to be pulled through the trachea cannula. Therefore, another gastroscope biopsy forcep was inserted through the trachea cannula to firmly clamp the edge of the FB. Finally, we pulled out the FB together with the trachea cannula smoothly and successfully, and the FB was confirmed to be a 60 mL-syringe cap with a size of approximately 1.3 cm × 3 cm (Fig. 2). Spontaneous ventilation and proper sedation were maintained throughout the procedure. A repeat CT, obtained the next day, showed well inflation of the left lung.

## 3 Discussion

Most of the FBAs occur in children; however, in adults, FBA is always associated with unconsciousness or swallowing dysfunction which is common in NICU. FBA is a clinical emergency, especially in the NICU, which can aggravate respiratory symptoms and even endanger the lives of patients. With the development of bronchoscopy technology, most FBs can be removed using forceps, baskets, or balloon-tipped catheter.^[3]^ However, the diversity of FBs makes the removal challenging. We must choose the appropriate removal method in different situations to remove the FBs as quickly and smoothly as possible. In this study, we present the case using fiberoptic bronchoscope together with gastroscope biopsy forceps to remove the needle cap for the first time.

FBs similar to syringe cap have also been reported, but they all have a hole on one side and are successfully removed by balloon catheter technique.^[4,5]^ Syringe cap is first reported as FB in this study with a positive history. In addition, 2 features of CT imaging, in this case, maybe helpful for the diagnosis:

1. a cylindrical-like FB of moderate density with the highest-density point at the proximal end (CT value is about 300 Hu);
2. no gap between the FB and the tracheal wall due to its hollow structure opening at one end and the inhalation effect.

The removal of a syringe cap is challenging. First, there is no hole in the visible end of it to let the catheter go through. Second, when inhaled, the open end enters the bronchus first and its sidewall is closely attached to the bronchus resulting in the impossibility to clamp, and we could only carefully clamp the small prominent edge of the “+” mark. Finally, it is too large to be pulled through the trachea cannula. To achieve a firm grip, we used a gastroscope biopsy forceps to clamp the cap wall and when the cap was moved to relatively larger tracheal space above the carina and with a firm grip on the cap we removed it together with the trachea cannula.

Patients may be experiencing hypoxemia and metabolic acidosis at the onset and special life support techniques are required for some severe cases of respiratory failure or instable hemodynamics.^[1,6]^ The patient presented in this case showed asphyxia immediately after FBA and trachea intubation together with mechanical ventilation were temporary used to restore oxygen supply.

For patients with an acute onset, maintaining effective ventilation at the beginning and during the whole procedure of removal is the key to success. At the same time, we should choose an appropriate method to remove FB. For FBs that cannot be firmly clamped for the first time, we hope this will be a bit of good advice to combine the application of another biopsy forceps to hold FBs on the larger tracheal carina.

## Author contributions

**Resources:** Guangshan Hao, Zhengang Ji, Lusu Yao.

**Writing – original draft:** Guangshan Hao.

**Writing – review & editing:** Lina Tang, Jianxin Zhu, Lusu Yao.
